# Aβ_1‐42_‐induced Mitochondrial Dysfunction in Human Neuroblastoma M17 cells: Time‐dependent Impact on Mitochondria

**DOI:** 10.1002/alz70855_106561

**Published:** 2025-12-24

**Authors:** Dona P. W. Jayatunga, Eugene Hone, Ralph N Martins

**Affiliations:** ^1^ Centre of Excellence for Alzheimer's Disease Research & Care, perth, Western Australia, Sri Lanka; ^2^ Genetics and Molecular Biology Unit, Faculty of Applied Sciences, University of Sri Jayewardenepura, Nugegoda, Western Province, Sri Lanka; ^3^ Centre for Alzheimer's Disease Research and Care, WA 6009, Western Australia, Australia; ^4^ Alzheimer's Research Australia, Perth, Western Australia, Australia; ^5^ Centre of Excellence for Alzheimer's Disease Research and Care, School of Medical and Health Sciences, Edith Cowan University, Joondalup, Western Australia, Australia; ^6^ Macquarie University, Sydney, NSW, Australia

## Abstract

**Background:**

Alzheimer's disease (AD) is one of the most common neurodegenerative disorders that affects geriatric populations across the globe. Deposition of the Aβ peptide in the brain is considered a central event of AD pathology. Mitochondrial dysfunction is a characteristic feature of AD. The objective of the present study was to determine the time‐dependent effects of Aβ_1‐42_ toxicity in BE(2)‐M17 and its role in mitochondrial quality control mechanisms namely, mitochondrial dynamics, selective autophagy and mitochondrial biogenesis.

**Method:**

Oligomeric Aβ_1‐42_ was added to the cells for time points of 4, 8, 16, 24, 48 and 72 hours. At all these time points, cellular mitochondrial dehydrogenase activity, ATP and reactive oxygen species (ROS) levels were determined using MTS, CellTiter Glo and DCFDA assays, respectively. Western blots were used to quantify mitochondrial dynamics‐related proteins: OPA1, MFN2 and FIS1, mitophagy related proteins: p62, NDP52, OPTN, PINK1 and TIMM23 and mitobiogenesis related protein, PGC‐1α.

**Result:**

Increased ROS levels, attenuated ATP levels and fluctuations of mitochondrial quality control proteins in cells at the respective time intervals suggest altered mitochondrial quality control. Furthermore, compromised mitochondrial dynamics, mitophagy and mitobiogenesis suggest that chronic Aβ_1‐42_ exposure promotes mitochondrial dysfunction.

**Conclusion:**

Further studies are required to determine if the time‐dependent fluctuations of these mitochondrial mechanisms were due to adaptive cell responses against the Aβ_1‐42_ cytotoxicity.

